# Dosing levels of antipsychotics and mood stabilizers in bipolar disorder: A Nationwide cohort study on relapse risk and treatment safety

**DOI:** 10.1111/acps.13762

**Published:** 2024-10-02

**Authors:** Jonne Lintunen, Aleksi Hamina, Markku Lähteenvuo, Tapio Paljärvi, Antti Tanskanen, Jari Tiihonen, Heidi Taipale

**Affiliations:** ^1^ Department of Forensic Psychiatry University of Eastern Finland, Niuvanniemi Hospital Kuopio Finland; ^2^ Norwegian Centre for Addiction Research (SERAF) Institute of Clinical Medicine, University of Oslo Oslo Norway; ^3^ Department of Clinical Neuroscience Karolinska Institutet Stockholm Sweden; ^4^ Neuroscience Center University of Helsinki Helsinki Finland; ^5^ School of Pharmacy University of Eastern Finland Kuopio Finland

**Keywords:** adverse effects, antipsychotic agents, bipolar disorder, mood stabilizers, relapse

## Abstract

**Background:**

Finding effective treatment regimens for bipolar disorder is challenging, as many patients suffer from significant symptoms despite treatment. This study investigated the risk of relapse (psychiatric hospitalization) and treatment safety (non‐psychiatric hospitalization) associated with different doses of antipsychotics and mood stabilizers in persons with bipolar disorder.

**Methods:**

Individuals aged 15–65 with bipolar disorder were identified from Finnish national health registers in 1996–2018. Studied antipsychotics included olanzapine, risperidone, quetiapine, aripiprazole; mood stabilizers lithium, valproic acid, lamotrigine, and carbamazepine. Medication use was divided into three time‐varying dose categories: low, standard, and high. The studied outcomes were risk of psychiatric hospitalization (relapse) and the risk of non‐psychiatric hospitalization (treatment safety). Stratified Cox regression in within‐individual design was used.

**Results:**

The cohort included 60,045 individuals (mean age 41.7 years, SD 15.8; 56.4% female). Mean follow‐up was 8.3 years (SD 5.8). Of antipsychotics, olanzapine and aripiprazole were associated with a decreased risk of relapse in low and standard doses, and risperidone in low dose. The lowest adjusted hazard ratio (aHR) was observed for standard dose aripiprazole (aHR 0.68, 95% CI 0.57–0.82). Quetiapine was not associated with a decreased risk of relapse at any dose. Mood stabilizers were associated with a decreased risk of relapse in low and standard doses; lowest aHR was observed for standard dose lithium (aHR 0.61, 95% CI 0.56–0.65). Apart from lithium, high doses of antipsychotics and mood stabilizers were associated with an increased risk of non‐psychiatric hospitalization. Lithium was associated with a decreased risk of non‐psychiatric hospitalization in low (aHR 0.88, 95% CI 0.84–0.93) and standard doses (aHR 0.81, 95% CI 0.74–0.88).

**Conclusions:**

Standard doses of lithium and aripiprazole were associated with the lowest risk of relapse, and standard dose of lithium with the lowest risk of non‐psychiatric hospitalization. Quetiapine was not associated with decreased risk of relapse at any dose.

**Significant outcomes**

- Higher doses of antipsychotics or mood stabilizers were not associated with better treatment outcomes.
- Standard doses of lithium and aripiprazole were associated with the lowest risk of relapse.
- With increasing evidence indicating its poor effectiveness, the wide use of quetiapine should be thoroughly re‐evaluated.

**Limitations**

- A large nationwide cohort was utilized, but information on some important variables (such as data on psychosocial or neuromodulation treatments) was not available and could not be adjusted for in this study.
- Information on the severity of symptoms was not available, and the hospitalization‐based outcomes can be imprecise as indicators of clinical status or treatment safety.

## 1 INTRODUCTION

Bipolar disorder is a severe psychiatric illness affecting approximately 2% of the population.^1^, ^2^ Despite pharmacological and psychosocial interventions, a large proportion of patients continue to experience symptoms that impair their quality of life and functioning. Individuals with bipolar disorder experience clinically meaningful symptoms approximately half of the time, and depressive symptoms are more common than hypomanic/manic symptoms.^3^, ^4^ Relapses are common during treatment; a UK study showed that around 25% of individuals with bipolar disorder relapsed over a 5‐year period in a cohort of people treated in secondary mental health services.^5^ Additionally, individuals with bipolar disorder are at significant risk of self‐harm. A lifetime prevalence of suicide attempt in bipolar disorder is over 30% and up to 15%–20% of persons with bipolar disorder die as a result of suicide.^6^

Pharmacological treatment for bipolar disorder usually includes mood stabilizers (such as lithium, valproic acid, lamotrigine, and carbamazepine) and antipsychotics (such as risperidone, olanzapine, quetiapine, aripiprazole).^1^, ^7^, ^8^ A common challenge in pharmacotherapy is finding a combination of medicines and doses that improve symptoms but do not cause significant adverse effects. A previous study found an increased risk of mortality among individuals using antipsychotics for bipolar disorder, whereas those prescribed mood stabilizers had a decreased mortality risk.^9^ The observed risk increase during antipsychotic treatment was dose‐dependent highlighting the importance of careful dose titration.

The aim of this study was to investigate which doses of antipsychotics and mood stabilizers are associated with a decreased risk of relapse without significantly increasing the risk of non‐psychiatric hospitalization in bipolar disorder.

## 2 METHODS

### 2.1 Study population

Those diagnosed with bipolar disorder (International Statistical Classification of Diseases and Related Health Problems, Tenth Revision (ICD‐10) codes F30‐F31) at the age of 15–65 years during 1987–2018 in Finland were included in this study. Four nationwide registers were used to identify the study population: sickness absence register (maintained by the Social Insurance Institution, data from 2004 to 2018), disability pension register (maintained by the Finnish Centre for Pensions, data from 1996 to 2018), inpatient care register and specialized outpatient care register (maintained by the National Institute of Health and Welfare, inpatient care register included data from 1987 to 2018 and outpatient care register from 1998 to 2018). Unique personal identification codes that are assigned to each person in Finland were used to link individual data from different registers. Individuals diagnosed with a schizophrenia‐spectrum disorder (ICD‐10: F20‐F29) or dementia (F00‐F03, G30) before they were diagnosed with bipolar disorder were excluded. The follow‐up time started from the cohort entry (first‐time diagnosis or January 1, 1996, whichever occurred first) and lasted until death, diagnosis of schizophrenia‐spectrum disorder, or the end of data linkage (December 31, 2018).

### 2.2 Exposures

The four most commonly used oral antipsychotics and mood stabilizers in the cohort were studied. Antipsychotics included olanzapine (Anatomical Therapeutic Chemical Classification System (ATC) code N05AH03), quetiapine (N05AH04), risperidone (N05AX08), and aripiprazole (N05AX12). Mood stabilizers included carbamazepine (N03AF01), valproic acid (N03AG01), lamotrigine (N03AX09), and lithium (N05AN01). Data on drug purchases was derived from the nationwide prescription drug register, which is maintained by the Social Insurance Institution. Drug purchase data was available from 1995 to 2018.

Drug use periods were calculated from the data on drug purchases using the PRE2DUP method. The method has been described in detail previously.^10^ In short, the PRE2DUP method models time periods when a person is using a specific medication based on information on the date of drug dispensing and amounts dispensed from pharmacies. In addition, PRE2DUP takes into account hospital stays, regularity of medication purchases and possible stockpiling of drugs. Each drug package has been assigned expert‐defined values of upper and lower limits for daily dose and the calculation are made in defined daily doses (DDDs). DDD is the assumed average maintenance dose per day for a medication used for its main indication in adults.^11^

The studied medications were further divided into three time‐varying dose‐categories based on DDDs (low: <0.9 DDDs, standard: 0.9‐<1.1 DDDs, and high: ≥1.1 DDDs). Reference values for valproic acid, lamotrigine and carbamazepine were changed so that they represented their use in bipolar disorder, since the original DDD values for valproic acid, lamotrigine and carbamazepine are for the treatment of epilepsy. Used reference values corresponding to 1 DDD were 1000 mg for valproic acid, 400 mg for carbamazepine, 200 mg for lamotrigine, and 900 mg for lithium.

### 2.3 Outcomes

The studied outcome measures were psychiatric hospitalizations (hospitalizations with ICD‐10 codes F00‐F99) indicating relapse, and non‐psychiatric hospitalizations (ICD‐10 codes other than F00‐F99) as an indicator of overall safety.

### 2.4 Statistical analyses

The analyses were conducted in within‐individual design, where each person forms their own stratum and time periods when a person is using a specific drug is compared with those time periods when the drug is not used. Since comparisons are made within the same individual, all time‐invariant covariates are controlled for by the study design. The analyses were adjusted for time‐varying covariates (time since cohort entry, age, temporal order of treatments, and the use of other psychotropic medications [antidepressants (N06A) and benzodiazepines and related drugs (N05BA, N05CD, and N05CF)]).

Stratified Cox regression models in within‐individual design were used to analyze the data. Antipsychotics were compared with non‐use of antipsychotics and mood stabilizers with non‐use of mood stabilizers. In both drug groups, polytherapy including multiple drugs from the same group was categorized separately and the results reported here represent monotherapy use. Hospitalization‐based outcomes were analyzed as recurrent events, meaning that they could occur multiple times for the same individual and the follow‐up time was reset to zero after each outcome event. In this way, it was possible to compare outcome rates during the use of different medicines within the same individual. Those individuals that had both treatment and non‐treatment periods and experienced an outcome event during the follow‐up directly contributed to the analyses. The rest of the cohort contributed to the estimates indirectly through time‐varying covariates.

In addition, to minimize the effects of an already discontinued medication during a treatment change, sensitivity analyses in which 30 days after each medication change were omitted were conducted.

## 3 RESULTS

The study cohort included 60,045 individuals (mean age 41.7 years, SD 15.8, 56.4% female), and the mean follow‐up time was 8.3 years (SD 5.8) (Table 1). At the cohort entry, 24.3% of the cohort had been hospitalized due to bipolar disorder. Anxiety disorder was the most common psychiatric comorbidity (26.6%). Mood stabilizer monotherapies were used by 32,345 persons in low‐dose, 18,174 in standard‐dose, and 20,933 in high‐dose (Table 2). Of high‐dose mood stabilizer users (*N* = 20,933), 90.5% (*N* = 18,936) had non‐use periods of mood stabilizers, 88.6% (*N* = 18,552) had low‐dose mood stabilizer use and 71.0% (*N* = 14,866) had standard‐dose mood stabilizer use during the follow‐up. Specific antipsychotics (olanzapine, quetiapine, risperidone, and aripiprazole) were used by 36,538 persons in low‐dose, 12,902 persons in standard‐dose, and 16,292 persons in high‐dose. Of high‐dose antipsychotic users (*N* = 16,292), 91.1% (*N* = 14,846) had antipsychotic non‐use periods, 92.2% (*N* = 15,026) had antipsychotic low‐dose use, and 58.6% (*N* = 9550) had antipsychotic standard‐dose use during the follow‐up.

**TABLE 1:** Characteristics of the study population at cohort entry.

Study population, *N*	60,045
Mean age, years (SD)	41.7 (15.8)
Females (*N*, %)	33,859 (56.4)
Males (*N*, %)	26,186 (43.6)
Previous hospitalization due to bipolar disorder at baseline (*N*, %)	14,600 (24.3)
Calendar year of index diagnosis (*N*, %)	
<2000	8452 (14.1)
2000–2009	26,744 (44.5)
2010–2018	24,849 (41.4)
Psychiatric comorbidities (*N*, %)	
Anxiety disorder	15,965 (26.6)
Personality disorder	8502 (14.2)
Substance use disorder	11,397 (19.0)
Previous suicide attempt	3345 (5.6)

Abbreviation: SD = standard deviation.

**TABLE 2:** Number of mood stabilizer (MS) and antipsychotic (AP) users and relapse events by dose categories.

	Dose	Events	Users	Person‐years
Mood stabilizers				
Any MS monotherapy use	<0.9	16,940	32,345	82,528
	0.9‐ < 1.1	5183	18,174	29,313
	≥1.1	13,554	20,933	39,235
Carbamazepine	<0.9	903	2100	3561
	0.9‐ < 1.1	366	988	1532
	≥1.1	1085	1296	3110
Valproic acid	<0.9	7203	15,422	30,450
	0.9‐ < 1.1	2234	7978	10,550
	≥1.1	6633	10,233	16,668
Lamotrigine	<0.9	4345	14,875	25,361
	0.9‐ < 1.1	1144	6312	9025
	≥1.1	2480	7123	8838
Lithium	<0.9	4489	9079	23,156
	0.9‐ < 1.1	1439	4938	8205
	≥1.1	3356	5523	10,619
Antipsychotics				
Any AP monotherapy use	<0.9	18,573	36,538	92,117
	0.9‐ < 1.1	2303	12,902	9418
	≥1.1	8001	16,292	18,381
Olanzapine	<0.9	2586	8317	12,153
	0.9‐ < 1.1	841	4690	3376
	≥1.1	3068	5950	6228
Quetiapine	<0.9	12,743	28,840	66,371
	0.9‐ < 1.1	1164	6607	4589
	≥1.1	4167	10,282	10,652
Risperidone	<0.9	2783	7392	11,274
	0.9‐ < 1.1	109	509	166
	≥1.1	180	740	232
Aripiprazole	<0.9	461	2683	2319
	0.9‐ < 1.1	189	2423	1287
	≥1.1	586	1743	1269

### 3.1 Risk of relapse, antipsychotics

**FIGURE 1:**
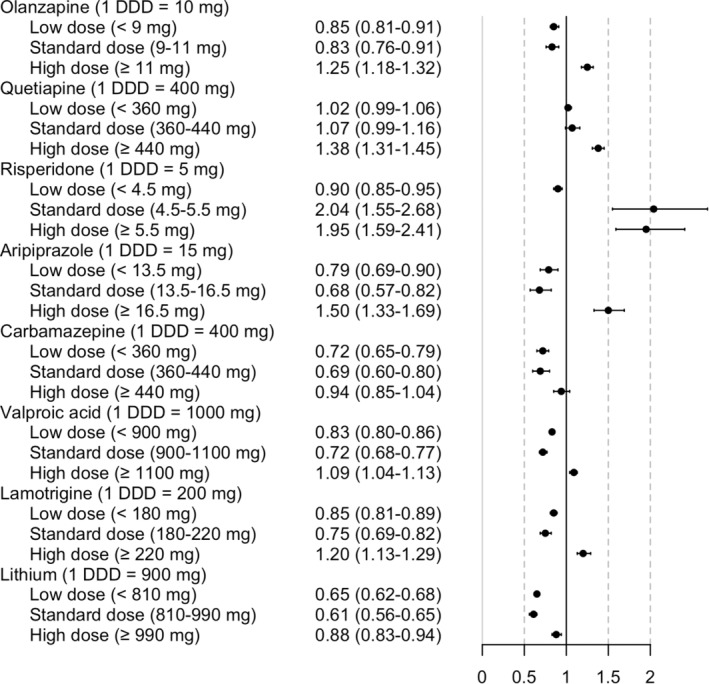
Risk of relapse in bipolar disorder during the use of antipsychotics or mood stabilizers in low (<0.9 DDDs), standard (0.9‐<1.1 DDDs), and high (≥1.1 DDDs) doses. Antipsychotics were compared with non‐use of antipsychotics and mood stabilizers with non‐use of mood stabilizers. Adjusted hazard ratios with 95% confidence intervals are shown.

As shown in Figure 1, olanzapine and aripiprazole were associated with a decreased risk of relapse in low doses (adjusted hazard ratio (aHR) with 95% confidence intervals (CIs) for low dose olanzapine aHR 0.85, 95% CI 0.81–0.91; aripiprazole aHR 0.79, 95% CI 0.69–0.90) and in standard doses (olanzapine aHR 0.83, 95% CI 0.76–0.91; aripiprazole aHR 0.68, 95% CI 0.57–0.82). Risperidone was associated with a decreased relapse risk only in low dose (aHR 0.90, 95% CI 0.85–0.95). The lowest aHR of antipsychotics was observed for standard dose aripiprazole. Quetiapine had no association with relapse risk in low (aHR 1.02, 95% CI 0.99–1.06) or standard doses (aHR 1.07, 95% CI 0.99–1.16). All four antipsychotics were associated with an increased risk of relapse in high doses (high‐dose risperidone aHR 1.95, 95% CI 1.59–2.41; high‐dose aripiprazole aHR 1.50, 95% CI 1.33–1.69; high‐dose quetiapine aHR 1.38, 95% CI 1.31–1.45; high‐dose olanzapine aHR 1.25, 95% CI 1.18–1.32). The use of standard dose risperidone had the highest aHR (aHR 2.04, 95% CI 1.55–2.68).

In the sensitivity analysis in which 30 days after each medication change were omitted, the use of low and standard doses of aripiprazole was associated with decreased relapse risk (low‐dose aripiprazole aHR 0.85, 95% CI 0.73–0.98; standard‐dose aripiprazole aHR 0.71, 95% CI 0.57–0.90) (Table 3). Each antipsychotic was associated with an increased risk of relapse in high doses (high‐dose aripiprazole aHR 1.49, 95% CI 1.29–1.72; high‐dose risperidone aHR 1.42, 95% CI 1.02–1.99; high‐dose quetiapine aHR 1.31, 95% CI 1.23–1.39; high‐dose olanzapine aHR 1.26, 95% CI 1.18–1.34). The use of quetiapine was associated with an increased risk of relapse also in low and standard doses (low‐dose quetiapine aHR 1.07, 95% CI 1.03–1.11; standard‐dose quetiapine aHR 1.13, 95% CI 1.03–1.24).

**TABLE 3:** Risk of relapse in bipolar disorder during the use of antipsychotics or mood stabilizers in low (<0.9 DDDs), standard (0.9‐<1.1 DDDs), and high (≥1.1 DDDs) doses when 30 days after each medication change were omitted. Antipsychotics were compared with non‐use of antipsychotics and mood stabilizers with non‐use of mood stabilizers. Adjusted hazard ratios with 95% confidence intervals are shown.

	Dose	aHR	95% CI
Olanzapine	<9 mg	0.98	0.92–1.04
	9–11 mg	0.90	0.81–1.00
	≥11 mg	1.26	1.18–1.34
Quetiapine	<360 mg	1.07	1.03–1.11
	360–440 mg	1.13	1.03–1.24
	≥440 mg	1.31	1.23–1.39
Risperidone	<4.5 mg	0.96	0.90–1.03
	4.5–5.5 mg	1.88	1.31–2.69
	≥5.5 mg	1.42	1.02–1.99
Aripiprazole	<13.5 mg	0.85	0.73–0.98
	13.5–16.5 mg	0.71	0.57–0.90
	≥16.5 mg	1.49	1.29–1.72
Carbamazepine	<360 mg	0.82	0.73–0.91
	360–440 mg	0.81	0.69–0.95
	≥440 mg	1.02	0.92–1.13
Valproic acid	<900 mg	0.92	0.88–0.96
	900–1100 mg	0.78	0.73–0.84
	≥1100 mg	1.13	1.07–1.18
Lamotrigine	<180 mg	0.90	0.85–0.95
	180–220 mg	0.78	0.71–0.86
	≥220 mg	1.23	1.14–1.33
Lithium	<810 mg	0.77	0.73–0.81
	810–990 mg	0.72	0.66–0.78
	≥990 mg	0.96	0.90–1.02

Abbreviations: aHR, adjusted hazard ratio; 95% CI, 95% confidence interval.

### 3.2 Risk of non‐psychiatric hospitalization, antipsychotics

**FIGURE 2:**
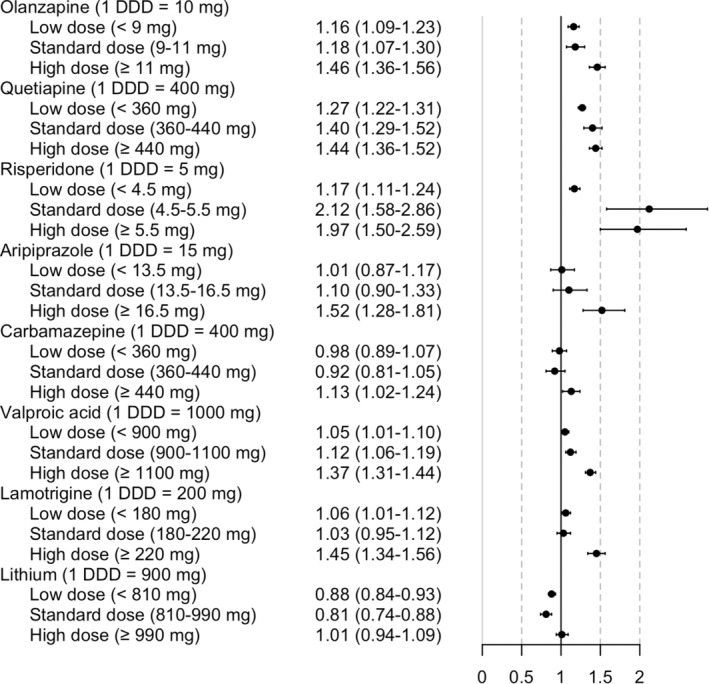
Risk of non‐psychiatric hospitalization in bipolar disorder during the use of antipsychotics or mood stabilizers in low (<0.9 DDDs), standard (0.9‐<1.1 DDDs), and high (≥1.1 DDDs) doses. Antipsychotics were compared with non‐use of antipsychotics and mood stabilizers with non‐use of mood stabilizers. Adjusted hazard ratios with 95% confidence intervals are shown.

Olanzapine, quetiapine and risperidone were associated with an increased risk of non‐psychiatric hospitalization in all dose categories (olanzapine low‐dose aHR 1.16, 95% CI 1.09–1.23, olanzapine standard‐dose aHR 1.18, 95% CI 1.07–1.30, olanzapine high‐dose aHR 1.46, 95% CI 1.36–1.56; quetiapine low‐dose aHR 1.27, 95% CI 1.22–1.31, quetiapine standard‐dose aHR 1.40, 95% CI 1.29–1.52, quetiapine high‐dose aHR 1.44, 95% CI 1.36–1.52; risperidone low‐dose aHR 1.17, 95% CI 1.11–1.24, risperidone standard‐dose 2.12, 95% CI 1.58–2.86, risperidone high‐dose aHR 1.97, 95% CI 1.50–2.59) (Figure 2). Aripiprazole was associated with an increased risk of non‐psychiatric hospitalization only in high doses (high‐dose aripiprazole aHR 1.52, 95% CI 1.28–1.81).

In the 30 days omission analysis all antipsychotics were associated with an increased risk of non‐psychiatric hospitalization in high doses (high‐dose risperidone aHR 1.68, 95% CI 1.22–2.33; high‐dose olanzapine aHR 1.39, 95% CI 1.29–1.50; high‐dose aripiprazole aHR 1.37, 95% CI 1.12–1.66; high‐dose quetiapine aHR 1.30. 95% CI 1.22–1.39) (Table 4). The use of quetiapine was associated with an increased risk of non‐psychiatric hospitalization also in low and standard doses (low‐dose aHR 1.19, 95% CI 1.15–1.24; standard‐dose aHR 1.20, 95% CI 1.09–1.32). Olanzapine and risperidone were associated with an increased risk of non‐psychiatric hospitalization in low doses (low‐dose olanzapine aHR 1.07, 95% CI 1.01–1.15; low‐dose risperidone aHR 1.12, 95% CI 1.05–1.19). Aripiprazole was associated with an increased risk of non‐psychiatric hospitalization only in high dose.

**TABLE 4:** Risk of non‐psychiatric hospitalization in bipolar disorder during the use of antipsychotics or mood stabilizers in low (<0.9 DDDs), standard (0.9‐<1.1 DDDs), and high (≥1.1 DDDs) doses when 30 days after each medication change were omitted. Antipsychotics were compared with non‐use of antipsychotics and mood stabilizers with non‐use of mood stabilizers. Adjusted hazard ratios with 95% confidence intervals are shown.

	Dose	aHR	95% CI
Olanzapine	<9 mg	1.07	1.01–1.15
	9–11 mg	1.00	0.89–1.13
	≥11 mg	1.39	1.29–1.50
Quetiapine	<360 mg	1.19	1.15–1.24
	360–440 mg	1.20	1.09–1.32
	≥440 mg	1.30	1.22–1.39
Risperidone	<4.5 mg	1.12	1.05–1.19
	4.5–5.5 mg	1.39	0.90–2.13
	≥5.5 mg	1.68	1.22–2.13
Aripiprazole	<13.5 mg	0.97	0.82–1.16
	13.5–16.5 mg	1.04	0.82–1.31
	≥16.5 mg	1.37	1.12–1.66
Carbamazepine	<360 mg	1.03	0.93–1.13
	360–440 mg	0.91	0.79–1.05
	≥440 mg	1.11	1.00–1.23
Valproic acid	<900 mg	1.10	1.05–1.15
	900–1100 mg	1.14	1.07–1.22
	≥1100 mg	1.35	1.28–1.42
Lamotrigine	<180 mg	1.11	1.05–1.18
	180–220 mg	1.05	0.96–1.15
	≥220 mg	1.34	1.23–1.47
Lithium	<810 mg	0.98	0.93–1.04
	810–990 mg	0.85	0.78–0.93
	≥990 mg	1.07	0.98–1.16

Abbreviations: aHR, adjusted hazard ratio; 95% CI, 95% confidence interval.

### 3.3 Risk of relapse, mood stabilizers

Mood stabilizers were associated with a decreased risk of relapse in low (carbamazepine aHR 0.72, 95% CI 0.65–0.79; valproic acid aHR 0.83, 95% CI 0.80–0.86; lamotrigine aHR 0.85, 95% CI 0.81–0.89; lithium aHR 0.65, 95% CI 0.62–0.68) and standard doses (carbamazepine aHR 0.69, 95% CI 0.60–0.80; valproic acid aHR 0.72, 95% CI 0.68–0.77; lamotrigine aHR 0.75, 95% CI 0.69–0.82; lithium aHR 0.61, 95% CI 0.56–0.65) (Figure 1). The lowest aHRs were observed for standard doses and standard‐dose lithium had the largest observed risk decrease (aHR 0.61, 95% CI 0.56–0.65). High‐dose lamotrigine and high‐dose valproic acid were associated with an increased relapse risk (lamotrigine aHR 1.20, 95% CI 1.13–1.29; valproic acid aHR 1.09, 95% CI 1.04–1.13). Lithium was the only mood stabilizer that was associated with a decreased risk of relapse in high doses (aHR 0.88, 95% CI 0.83–0.94).

In the 30 days omission analysis all mood stabilizers were associated with a decreased risk of relapse in low and standard doses (Table 3). The use of standard‐dose lithium was associated with the lowest aHR (aHR 0.72, 95% CI 0.66–0.78). High‐dose valproic acid and lamotrigine were associated with an increased risk of relapse (high‐dose valproic acid aHR 1.13, 95% CI 1.07–1.18; high‐dose lamotrigine aHR 1.23, 95% CI 1.14–1.33). High‐dose carbamazepine and high‐dose lithium were not associated with relapse risk (carbamazepine aHR 1.02, 95% CI 0.92–1.13; lithium aHR 0.96, 95% CI 0.90–1.02).

### 3.4 Risk of non‐psychiatric hospitalization, mood stabilizers

Carbamazepine was associated with an increased risk of non‐psychiatric hospitalization in high doses (aHR 1.13, 95% CI 1.02–1.24), valproic acid in all dose categories (low‐dose aHR 1.05, 95% CI 1.01–1.10; standard‐dose aHR 1.12, 95% CI 1.06–1.19; high‐dose aHR 1.37, 95% CI 1.31–1.44), and lamotrigine in low and high doses (low‐dose aHR 1.06, 95% CI 1.01–1.12; high‐dose aHR 1.45, 95% CI 1.34–1.56) (Figure 2). Lithium was not associated with an increased risk, but low and standard doses were associated with a decreased risk of non‐psychiatric hospitalization (low dose aHR 0.88, 95% CI 0.84–0.93; standard dose aHR 0.81, 95% CI 0.74–0.88).

When 30 days after each medication change were omitted, the use of valproic acid was associated with an increased risk of non‐psychiatric hospitalization in all dose categories (low‐dose aHR 1.10, 95% CI 1.05–1.15; standard‐dose aHR 1.14, 95% CI 1.07–1.22; high‐dose aHR 1.35, 95% CI 1.28–1.42) (Table 4). Lamotrigine was associated with an increased risk of non‐psychiatric hospitalization in low and high doses (low‐dose aHR 1.11, 95% CI 1.05–1.18; high‐dose aHR 1.34, 95% CI 1.23–1.47). Carbamazepine had no association on the risk of non‐psychiatric hospitalization, and the use of standard‐dose lithium was associated with a decreased risk (aHR 0.85, 95% CI 0.78–0.93).

## 4 DISCUSSION

This study investigated the risk of relapse and treatment safety associated with specific dosing levels of antipsychotics and mood stabilizers in persons with bipolar disorder.

In most of the studied medications, standard doses of medications were associated with the lowest risk of relapse, measured by recurrent psychiatric hospitalizations. The same applied also to non‐psychiatric hospitalizations, which were used as a proxy measure to adverse effects. Therefore, it appears that standard doses may be both the most effective and safest treatment choice in bipolar disorder.

Of antipsychotics, the use of standard‐dose aripiprazole was associated with the lowest risk of relapse supporting its use in bipolar disorder. In addition, the use of aripiprazole was not associated with an increased risk of non‐psychiatric hospitalization in low and standard doses. In previous studies, aripiprazole has shown efficacy in acute mania and in the prevention of mania, but its efficacy in depressive symptoms of bipolar disorder has been uncertain.^12^ This is problematic, since depressive episodes are more common than manic or hypomanic episodes in bipolar disorder.^8^ In two randomized, placebo‐controlled trials (RCTs), aripiprazole did not significantly reduce the depressive symptoms of bipolar disorder.^13^ However, a post hoc analysis of these two RCTs suggested that aripiprazole could be effective for individuals with more severe depressive symptoms, particularly at a lower dose (5–15 mg/day).^14^ Overall, these results support the use of aripiprazole in bipolar disorder in low and standard doses, but the use of higher doses (>15 mg/day) should be carefully considered. Other second‐generation oral antipsychotics that were associated with a decreased risk of psychiatric hospitalization in low dose were olanzapine and risperidone, and the use of olanzapine was also associated with a decreased risk of relapse in standard dose. However, the use of olanzapine and risperidone in low and standard doses was associated with an increased risk of non‐psychiatric hospitalization.

Based on the results of this study, quetiapine does not seem to be effective in the treatment of bipolar disorder. Furthermore, quetiapine was associated with an increased risk of non‐psychiatric hospitalization in all dose categories. This is significant, considering that quetiapine is often recommended for bipolar disorder in national and international guidelines.^15^, ^16^, ^17^ It is commonly used in bipolar disorder,^18^ even though there is increasing evidence of its poor effectiveness.^18^, ^19^, ^20^, ^21^ Our results are in line with these previous results and strengthen those, as we did not observe beneficial effect at any dose, and the risk of non‐psychiatric hospitalization was increased during quetiapine use. This also argues against the conception that the lack of observed effect in register‐based studies is related to the common low‐dose use of quetiapine.

All antipsychotics studied were associated with an increased risk of non‐psychiatric hospitalization. However, this association was not as strong when antipsychotics were used at low or standard doses. Physical comorbidities are common and individuals with bipolar disorder have an increased risk of mortality,^1^, ^9^, ^22^, ^23^ and some of that risk could be due to medications, especially at higher doses.^9^ Therefore, the use of higher doses should be carefully considered and, if possible, avoided. On the other hand, higher risk of non‐psychiatric hospitalization that was observed in this study may also reflect better monitoring of physical health problems (in high‐dose users) and signal that they have been properly treated.

Among mood stabilizers, lithium was associated with the lowest risk of psychiatric hospitalization. Additionally, an important finding was that none of the lithium doses showed a clear association with an increased risk of non‐psychiatric hospitalization. This is interesting considering lithium's well‐known risk for severe, albeit rare somatic adverse effects, such as reduced renal function, hyperparathyroidism, and lithium toxicity.^1^, ^8^, ^24^ It is possible that lithium's efficacy in bipolar disorder also protects against somatic illnesses, as individual's mental well‐being is more stable during lithium use, enabling them to better care for themselves. Moreover, regular blood monitoring, which is obligatory during lithium use, may lead to early detection of somatic illnesses, thus reducing the likelihood of hospitalization due to complications from somatic diseases. Additionally, lithium is not associated with as significant metabolic health issues, such as weight gain^25^ as, for example, valproate.^26^ This may result in a relatively lower risk of developing cardiovascular diseases or other common comorbidities associated with other medications that are used in bipolar disorder. Lithium has been shown to be the most effective medication in the maintenance treatment of bipolar disorder preventing both depressive and manic episodes.^27^ Despite this, lithium is still prescribed relatively rarely, and the use of lithium has significantly declined over the past decades.^28^, ^29^ Thus, lithium should be used more often in clinical practice. The use of carbamazepine was also associated with a reduced risk of relapse at low and standard doses without an increase in non‐psychiatric hospitalization risk. However, the use of carbamazepine is limited by its interactions with other psychotropic medications, and therefore, it is not recommended as a first‐line treatment in the Finnish treatment guidelines.^16^

This study has both strengths and limitations. It utilized a large, nationwide cohort with data obtained from multiple registers. Register data in Finland is known to be of high quality,^30^ making it suitable for pharmacoepidemiological research.^31^ One significant strength of this study was the use of a within‐individual design, which minimizes common statistical biases associated with register‐based research, since this study design controls for all time‐invariant factors. Additionally, important time‐varying variables were controlled for statistically. However, this study included only Finnish individuals, so the results may not be directly generalizable to other countries that have, for example, different healthcare systems. Another limitation is the lack of clinical variables on patient characteristics, such as information on the severity of illness and laboratory test results. Medication concentration measurements were not included in the registers, which is a major limitation, especially concerning lithium. Psychosocial treatments, such as psychotherapies, and neuromodulation treatments could not be adjusted for in this study. The primary outcomes of this study were hospitalizations, and information on the severity of self‐reported symptoms or quality of life were not available. Additionally, we studied the risk of psychiatric hospitalization in general and did not differentiate between, for example, manic and depressive episodes, as this would have reduced statistical power significantly. Similarly, the risk of non‐psychiatric hospitalization was studied in general, which can be regarded as a somewhat imprecise metric of treatment safety. Non‐psychiatric hospitalization may signal treatment safety, but it may also describe the intensity of efforts to prevent and treat physical health problems during medication use. It should be noted that psychotropics have a rather long onset of action, so hospitalizations that occurred within the first few weeks of treatment may not always indicate ineffectiveness. To minimize this limitation, a sensitivity analysis in which 30 days after each medication change were omitted was conducted. The results of this sensitivity analysis were similar to those of the main analysis; the use of quetiapine at any dose was not associated with a decreased relapse risk (instead the risk was increased in all dose categories), low and standard doses of aripiprazole were associated with a decreased relapse risk, and low and standard doses of lithium were associated with a decreased relapse risk. However, high dose of lithium was not associated with a statistically significant decreased or increased relapse risk in the sensitivity analysis (aHR 0.96, 95% CI 0.90–1.02). As the results are indicative of a protective effect, it is possible that this difference in results is due to decreased statistical power following decreased exposure periods when each change in medication leads to an omission of 30 days. It should also be noted that individuals with particularly severe bipolar disorder may be more likely to be prescribed higher medication doses, which could overestimate the risk of relapse associated with high doses. However, most of the high‐dose users had also time periods when they were using small and standard doses and the analyses were made in within‐individual design, which minimizes this limitation compared to, for example, traditional between‐individual analyses.

In summary, based on the results of this register‐based cohort study, higher doses of antipsychotics or mood stabilizers were not associated with better treatment outcomes. Instead, standard doses were associated with lower risks of both relapse and non‐psychiatric hospitalizations. Of antipsychotics, standard‐dose aripiprazole was associated with the lowest risk of relapse. Further research on its use in bipolar disorder, especially in bipolar depression, is needed. Additionally, the wide use quetiapine in the treatment of bipolar disorder should be thoroughly re‐evaluated, as there is increasing evidence indicating its poor effectiveness and increased risk of adverse events regardless of dose. In line with previous research findings, lithium was the most effective medication for bipolar disorder, and it should be used more frequently.

## FUNDING INFORMATION

The study was partially funded by the Ministry of Social Affairs and Health, Finland, through the developmental fund for Niuvanniemi Hospital (funding number 330104). The funder of the study had no role in study design, data collection, data analysis, data interpretation, or the writing of the report.

## CONFLICT OF INTEREST STATEMENT

J.T., H.T., and A.T. have participated in research projects funded by grants from Janssen‐Cilag and Eli Lilly to their employing institution. H.T. reports personal fees from Gedeon Richter, Janssen‐Cilag, Lundbeck and Otsuka. J.T. has been a consultant to HLS Therapeutics, Orion, and WebMed Global, and has received honoraria from Eli Lilly, Evidera, Janssen‐Cilag, Lundbeck, Mediuutiset, Otsuka, Sidera, and Sunovion. M.L. is a board member of Genomi Solutions Ltd. and Nursie Health Ltd., has received honoraria from Sunovion, Orion Pharma, Otsuka, Lundbeck, Recordati and Janssen‐Cilag. Other authors report no conflicts of interests.

## ETHICS STATEMENT

This study was approved by the Finnish National Institute for Health and Welfare (permission 635/5.05.00/2019), Finnish Centre for Pensions (19023), the Social Insurance Institution of Finland (31/522/2019), and Statistics Finland (TK‐53‐569‐19). Consent was not obtained from participants, since this is not required by the Finnish law in studies using pseudonymized administrative registers.

## Data Availability

The datasets analyzed in this study are not publicly available due to participant privacy and security concerns. Access to the data can be applied from the Finnish Social and Health Data Permit Authority (Findata).
